# Cardiovascular Autonomic Function and Progression of Age-Related Macular Degeneration in The Irish Longitudinal Study of Ageing (TILDA)

**DOI:** 10.1167/iovs.65.6.24

**Published:** 2024-06-14

**Authors:** Emma Connolly, Silvin P. Knight, Eoin Duggan, Siobhan Scarlett, Louise Newman, Mark Cahill, Rose Anne Kenny, Sarah L. Doyle, Roman Romero-Ortuno

**Affiliations:** 1 The Irish Longitudinal Study on Ageing, Trinity College Dublin, Dublin, Ireland; 2 Discipline of Medical Gerontology, School of Medicine, Trinity College Dublin, Dublin, Ireland; 3 Progressive Vision Research, Dublin, Ireland; 4 Department of Clinical Medicine, School of Medicine, Trinity College Dublin, Ireland; 5 Trinity College Institute of Neuroscience, Trinity College Dublin, Dublin, Ireland; 6 Global Brain Health Institute, Trinity College Dublin, Dublin, Ireland

**Keywords:** cardiovascular, autonomic, macular degeneration

## Abstract

**Purpose:**

To examine if changes in hemodynamic measures during an orthostatic challenge were associated with progression of age-related macular degeneration (AMD) over a 4-year period in The Irish Longitudinal Study on Ageing.

**Methods:**

Participants with AMD who underwent an active stand (AS) test at wave 1 (2009/2010) and retinal photographs at both wave 1 and wave 3 (2014/2015) were included (*N* = 159: 121 with no AMD progression and 38 with progression). Beat-to-beat hemodynamic data were non-invasively collected using a Finometer MIDI device during the AS at wave 1, recording systolic blood pressure (sBP), diastolic blood pressure (dBP), mean arterial pressure (MAP), and heart rate. Cardiac output, stroke volume, and total peripheral resistance (TPR) were derived from these measures. Baseline characteristics were compared between groups with and without AMD progression. Mixed-effects linear regression models were used to assess the association between changes in hemodynamic parameters during the AS and AMD progression, controlling for known AMD-associated risk factors.

**Results:**

At baseline, increasing age and lower dBP were significantly associated with AMD progression. Mixed-effects models for the period between standing and 10 seconds post-stand revealed significant associations with AMD progression with a steeper drop in dBP and a slower drop in TPR. Between 10 and 20 seconds post-stand, AMD progression was significantly associated with less pronounced reduction in heart rate.

**Conclusions:**

These observational data suggest that impaired hemodynamic responses within the first 20 seconds of orthostasis may be associated with the progression of AMD.

The autonomic nervous system (ANS) plays a pivotal role in regulating physiological processes during standing up (orthostasis), where the gravity-induced redistribution of blood centrally to the lower limbs requires an instantaneous reaction from the ANS to maintain blood pressure and preserve homeostasis.^1^ This function, facilitated through the baroreceptor reflex, senses a drop in blood pressure (BP) upon standing and, through coordinated responses of the sympathetic and parasympathetic branches of the ANS, increases heart rate and vascular tone to stabilize BP.^2^

Age-related reductions in the functions of the autonomic and cardiovascular systems have been linked with increased prevalence of cardiovascular diseases (CVDs) and neurological impairments in older people.^3^^,^^4^ Age-related macular degeneration (AMD) is a progressive disease whose prevalence increases significantly with age. It is the leading cause of irreversible vision loss in people over 50 years of age in developed countries and accounts for 8.7% of all blindness worldwide,^5^ with its prevalence projected to increase significantly with the growing aging population.^5^^–^^8^

Several risk factors associated with AMD are also implicated in CVD, suggesting a shared pathophysiology between the two conditions.^9^ Although it is established that autonomic neurovascular dysregulation, in particular orthostatic hypotension (OH), can play a significant role in the development and progression of CVD,^10^ little is known about the influence of autonomic neurovascular dysfunction on AMD progression.

Impairment of neurovascular responses to actively standing can result in OH, causing dizziness, visual disturbances, and incidents of falls and syncope,^11^^–^^13^ and hypoperfusion of the cerebral vasculature during OH-related hypotension has been associated with increased risk of cognitive decline and dementia.^14^^,^^15^ This suggests that impaired orthostatic hemodynamics can contribute to end-organ damage. The eye, like the brain, is highly vascularized to support the neural retina and thus is potentially sensitive to OH-triggered blood flow impairment.

The active stand (AS) test with continuous non-invasive hemodynamic monitoring has emerged as a valuable tool for assessing autonomic function in clinical practice and research.^16^ During active transitions from a supine to an upright posture, continuous hemodynamic changes can be observed within the critical first 30 to 40 seconds post-stand, when most responses to counteract the movement of blood to the lower extremities take place to ensure adequate perfusion of vital organs.

In this retrospective observational study, we hypothesized that impaired orthostatic hemodynamics may be associated with the progression of AMD. In a subset of participants from The Irish Longitudinal Study on Ageing (TILDA), a well-defined sample of community-dwelling adults living with AMD in the Republic of Ireland, we compared hemodynamic responses to the AS test in participants with and without AMD progression over a 4-year period to determine if indicators of impaired cardiovascular autonomic function were associated with AMD progression during this time.

## Methods

### Study Population

This study utilized data from TILDA, a national cohort study established at Trinity College Dublin in 2009. TILDA collects health, social, and economic data through a series of data collection waves once every 2 years, with health assessments collecting physical and biological measurements occurring every 4 years. Selection and enrollment of participants in the first wave of TILDA were facilitated through a geodirectory-based random sampling procedure of residential addresses in the Republic of Ireland, resulting in a household response rate of 62%, as previously described.^17^ During the wave 1 data collection phase (2009–2011), AS data and retinal images were acquired during a comprehensive health assessment in a dedicated health center. Our analytical sample consisted of participants who had AMD at wave 1 and information on AMD progression at wave 3 approximately 4 years later, when a second retinal photograph was taken. For each wave of the study, ethical approval was granted by the Faculty of Health Sciences Research Ethics Committee at Trinity College Dublin, and all participants provided written informed consent. All experimental procedures were in accordance with the tenets of the Declaration of Helsinki.

### Retinal Photography and AMD Grading

Retinal photography was taken from participants during the health assessment at wave 1 and wave 3 by trained research nurses in TILDA. Images were acquired in wave 1 using a NIDEK AFC-210 fundus camera (NIDEK, Aichi, Japan) and in wave 3 using a NIDEK AFC-330 non-mydriatic auto fundus camera through a non-diluted pupil. Images were centered on the macula and included the optic disc (Early Treatment Diabetic Retinopathy Study [ETDRS] standard field 2). The grading system utilized was a modified version of the International Classification and Grading System for AMD^18^ with the age-related maculopathy (ARM) category replaced by three categories of early AMD: early mild (>10 hard macular drusen ≥ 63 µm), early moderate (at least one soft druse > 125 µm), and early severe (soft drusen and hyperpigmentation). Participants with late AMD were defined as late neovascular (choroidal neovascularization), late atrophic (geographic atrophy), or late-mixed (signs of late neovascular and late atrophic). Progression of AMD was determined by the same ophthalmologist through comparison of wave 1 and wave 3 images in the same participants and based on worst eye. Progression was defined as worsening to a higher stage of AMD.

### Orthostatic Challenge

Participants underwent an AS test with non-invasive beat-to-beat hemodynamic monitoring using a digital photoplethysmography-based Finometer MIDI device (Finapres Medical Systems, Arnhem, The Netherlands). The protocol required participants to lie supine for 10 minutes in a quiet room at ambient temperature. Participants were then asked to stand as fast as possible, unaided or with minimal assistance if required, and remain standing for 3 minutes while cardiovascular data were continuously monitored. Onset of the stand was detected using data from the Finometer height correction unit. Outputs from the Finometer included systolic BP (sBP), diastolic BP (dBP), heart rate (HR), and mean arterial pressure (MAP). The built-in Modelflow algorithm also allowed obtaining derived hemodynamic parameters including stroke volume (SV), cardiac output (CO), and total peripheral resistance (TPR).^19^ Baseline values were taken as the average reading between 60 and 30 seconds before standing. In this study, we utilized data up to 120 seconds post-stand, at 10-second intervals as per previously described methodology.^16^

### Additional Measures

Biological and demographic variables associated with the risk of AMD or which may influence hemodynamic changes were examined. These included age; sex; body mass index (BMI); waist-to-hip ratio; time taken to stand (stand duration); seated sBP/dBP; education status (primary, secondary, tertiary); geographical location (Dublin city/county, other urban/town, rural area); smoking status (never or past smoking, current smoking); Fried's frailty phenotype (not frail, pre-frail, frail)^20^; number of cardiovascular/neurovascular diseases (from this list: angina, heart attack, heart failure, stroke, transient ischemic attack, and heart murmur); being on antihypertensive medications (no/yes) as coded by the Anatomical Therapeutic Chemical (ATC) Classification (anti-arrhythmic [ATC C01], antihypertensives [ATC C02], diuretics [ATC C03], vasodilators [ATC C04], beta-blocking agents [ATC C07], calcium channel blockers [ATC C08], or agents acting on the renin–angiotensin system [ATC C09]); AMD phenotype at baseline; family history of AMD (no/don't know vs. yes); carriers of single nucleotide polymorphism (SNP) variants in genes encoding complement factor H (*CFH*) rs1061170; and age-related maculopathy susceptibility 2 (*ARMS2*) rs10490924.^21^^,^^22^

### Statistical Analysis

Descriptive statistics were performed using SPSS Statistics 28 (IBM Corporation, Chicago, IL, USA) and Stata 15.1 (StataCorp LLC, College Station, TX, USA). Continuous, non-normally distributed variables were summarized as means and standard deviations (SDs), and associations with AMD progression status were examined by the Mann–Whitney *U* test. Categorical variables are presented as counts and percentages, and associations with AMD progression were examined by χ^2^ test. Mixed-effects linear regression analysis was used to examine multiple repeated hemodynamic measures. Piece-wise linear splines were employed to model the time points at 0 to 10 seconds, 10 to 20 seconds, 20 to 30 seconds, 30 to 40 seconds, and 40 to 120 seconds post-stand. The linear splines were entered into the model as independent variables and as an interaction term with AMD progression status and covariates. Model 1 was adjusted for age and sex. Model 2 included age, sex, BMI, smoking status, number of cardiovascular/neurovascular conditions, use of antihypertensive medications, and AMD phenotype at baseline. Model 3, the fully adjusted model, included all of the previous variables, and carriers of the *CFH* and *ARMS2* risk alleles and a family history of AMD were included as main effects. To account for multiple comparisons in the mixed- effects models, a Bonferroni-corrected significance was set at *P* < 0.01 for association between AMD progression and BP measures across time points. The association between AMD-associated risk variables and BP measures at 0 to 10 seconds post-stand or 10 to 20 seconds post-stand employed a Bonferroni-corrected significance of *P* < 0.003.

## Results

**Figure 1.**
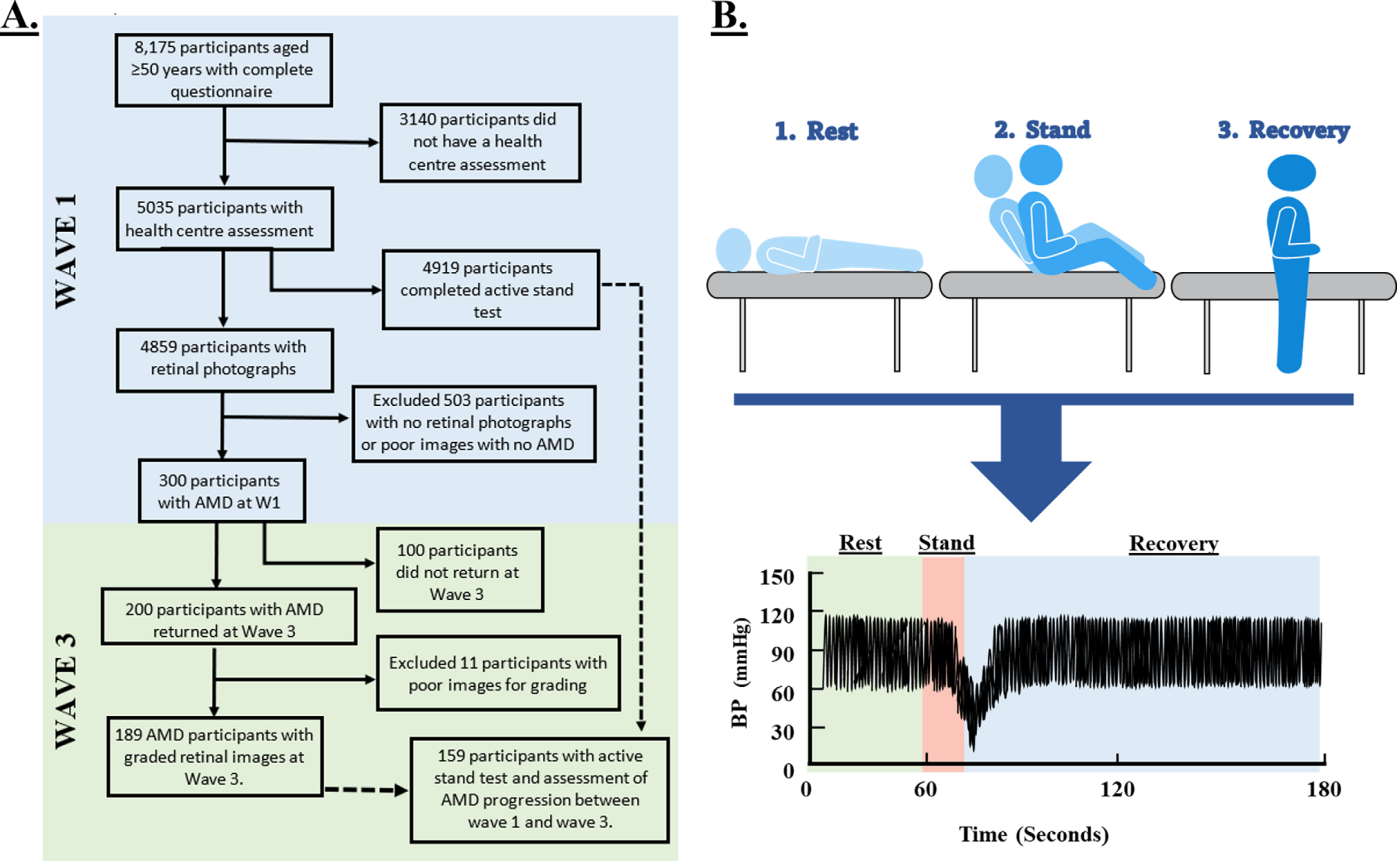
Flowchart of participant selection and schematic of active stand data protocol and data collection.

Figure 1 shows the flowchart of included participants (Fig. 1A) and a visual summary of the AS experiment (Fig. 1B). During the wave 1 health center assessment, 300 participants were found to have AMD. At the wave 3 health center assessment 4 years later, 200 participants returned for a follow-up assessment of AMD status, with 189 participants having gradable images. This information was matched with the AS data collected during the wave 1 health center assessment, revealing that 159 participants with AMD completed the AS test. Among them, 121 participants (76%) showed no progression of AMD, where 38 participants (24%) displayed progression of the disease. A breakdown of participants’ AMD status at wave 1 and change at wave 3 is presented in Supplementary Table S1.

Supplementary Table S2 reports the characteristics of participants who returned or did not return at wave 3. Of the 100 participants with AMD at wave 1 who did not have retinal images taken at wave 3, 65% completed the computer-aided personal interview (CAPI) but did not have images taken during the health center assessment, 29% did not take part in wave 3, and 6% had died between waves 1 and 3. Examination of wave 1 characteristics found that those who completed the CAPI but did not have images taken were significantly older (*P* = 0.008) and had higher seated sBP (*P* = 0.037) than participants who returned and had images taken. Participants who died between waves were significantly older (*P* = 0.006) and had significantly lower seated dBP (*P* = 0.028) than participants who returned at wave 3. There was no significant difference in these measures between participants who returned and those who chose not to take part in wave 3.

Table 1 presents a comparison of the characteristics of individuals with and without AMD progression. Older age was significantly associated with AMD progression (mean difference of 4 years; *P* = 0.015), and seated dBP was significantly lower in the progression group (*P* = 0.017). The proportion of current smokers was significantly higher in participants whose AMD progressed (*P* = 0.030), and antihypertensive use was also more frequent in this group (*P* = 0.035). Notably, the proportion of carriers of the Y402H polymorphism in CFH (rs1061170) was significantly higher in the AMD progression group, and family history of AMD was also more frequent in this group (*P* = 0.002). None of the other characteristics in Table 1 differed significantly between groups.

**Table 1.** Baseline Demographic Characteristics by AMD Progression Status

	AMD No Progression (*n* = 121)	AMD Progression (*n* = 38)	Total (*n* = 159)	*P*
Sex, *n* (%)				0.710
Male	50 (41.3)	17 (44.7)	67 (42.1)	
Female	71 (58.7)	21 (55.3)	92 (57.9)	
Age (y), mean (SD)	62 (9)	66 (9)	63 (9)	0.015^*^
BMI, mean (SD)	28.21 (4.75)	27.88 (3.96)	28.13 (4.56)	0.664
Waist-to-hip ratio, mean (SD)	0.90 (0.08)	0.90 (0.07)	0.90 (0.08)	0.865
Seated sBP, mean (SD)	135.50 (19.81)	133.88 (16.76)	135.10 (19.08)	0.622
Seated dBP, mean (SD)	83.94 (11.12)	79.15 (10.32)	82.78 (11.09)	0.017^*^
Pulse pressure, mean (SD)	51.55 (12.78)	54.72 (11.93)	52.32 (12.62)	0.166
Finometer baseline sBP, mean (SD)	140.03 (21.09)	135.20 (24.83)	138.87 (22.05)	0.283
Finometer baseline dBP, mean (SD)	75.11 (10.81)	70.27 (12.92)	73.95 (11.50)	0.041^*^
Finometer pulse pressure, mean (SD)	64.92 (14.70)	64.92 (16.41)	64.92 (15.07)	0.999
Stand duration (s), mean (SD)	7.3 (2.30)	8.13 (2.81)	7.49 (2.45)	0.099
Education, *n* (%)				0.662
Primary	18 (14.9)	8 (21.1)	26 (16.35)	
Secondary	50 (41.3)	15 (39.5)	65 (40.8)	
Tertiary	53 (43.8)	15 (39.5)	68 (42.7)	
Location, *n* (%)				0.527
Dublin city/county	36 (29.8)	8 (21.1)	44 (27.7)	
Other urban/town	32 (26.5)	10 (26.3)	42 (26.4)	
Rural area	53 (43.8)	20 (52.6)	73 (45.9)	
Smoking status, *n* (%)				0.030^*^
Never/past	113 (93.4)	31 (81.6)	144 (90.6)	
Current	8 (6.6)	7 (18.4)	15 (9.43)	
Employment, *n* (%)				0.077
Employed	55 (45.5)	10 (26.3)	65 (40.9)	
Retired	39 (32.2)	19 (50.0)	58 (36.5)	
Other	27 (22.3)	9 (23.7)	36 (22.6)	
Frailty status, *n* (%)				0.432
Not frail	83 (70.3)	27 (77.1)	110 (71.9)	
Pre-frail/frail	35 (29.7)	8 (22.9)	43 (28.1)	
CVD/NVD burden, *n* (%)				0.606
0	101 (83.5)	29 (76.3)	130 (81.8)	
1 or 2	18 (14.9)	8 (21.1)	26 (16.3)	
≥3	2 (1.6)	1 (2.6)	3 (1.9)	
Antihypertensives, *n* (%)				0.035^*^
No	86 (71.1)	20 (52.6)	106 (66.7)	
Yes	35 (28.9)	28 (47.4)	53 (33.3)	
*CFH (rs1061170), n (%)*				
Wild-type	40 (34.8)	4 (10.8)	44 (28.9)	0.005^*^
Carrier	75 (65.2)	33 (89.2)	108 (71.1)	
*ARMS2 (rs10490924), n (%)*				
Wild-type	60 (53.1)	15 (39.5)	75 (49.7)	0.146
Carrier	53 (46.9)	23 (60.5)	76 (50.3)	
Family history of AMD (yes), *n* (%)	5 (4.13)	8 (21.05)	13 (8.2)	0.002^*^

* Statistically significant.

**Figure 2.**
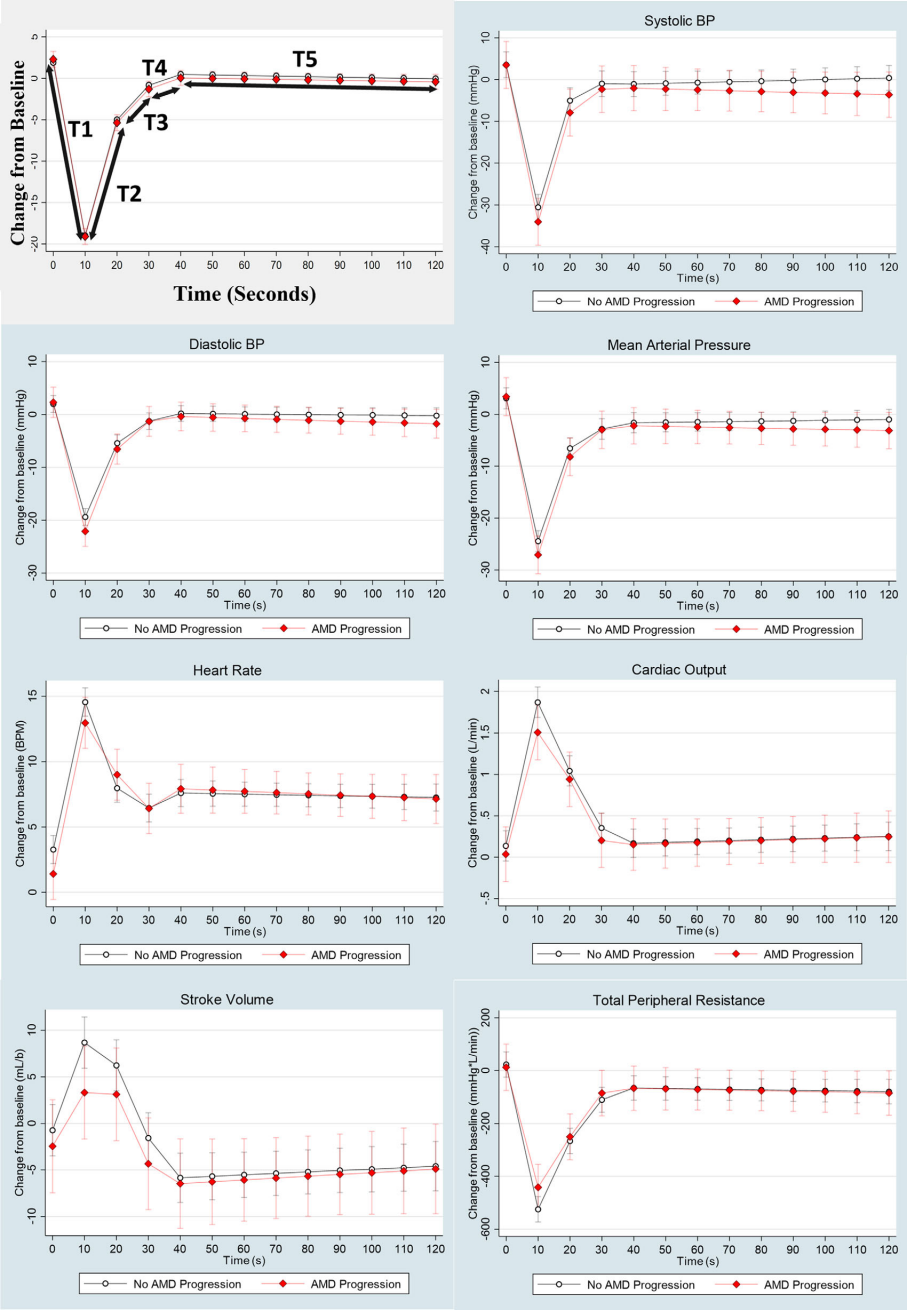
Change in hemodynamic measures in participants with or without AMD progression during the active stand test. Predicted means and 95% confidence intervals from mixed effect linear regression analysis for sBP, dBP, MAP, HR, CO, TPR, and SV are stratified by AMD progression status (AMD progression/no AMD progression). Model was adjusted for age, sex, BMI, smoking status, CVD burden, antihypertensive medication use, family history of AMD, and carriers of the *CFH* and *ARMS2* risk variant. Schematic shows placement of piecewise linear splines in the analysis.

Figure 2 visualizes the estimated marginal means with 95% confidence intervals from the fully adjusted mixed-effects linear regression models for changes in sBP, dBP, MAP, HR, CO, TPR, and SV during the AS, stratified by AMD progression status. As shown in Table 2, when controlling for covariates in the fully adjusted model, we found that progression of AMD was significantly associated with changes in dBP and TPR during the initial 0 to 10 seconds post-stand period (T1; see Fig. 1). This corresponded to a steeper drop in dBP (β_1_ *= −*0.302, *P* = 0.003) and a lesser decline in TPR (β_1_ = 9.357, *P* = 0.001) in the AMD progression group. Those with AMD progression also had a slower rate of HR stabilization (β_1_ = 0.262, *P* < 0.001) at 10 to 20 seconds post-stand compared to those with no AMD progression (Table 2).

**Table 2.** Change in Hemodynamic Measures at Time Points T1 to T5 Associated With AMD Progression Derived from Mixed-Effects Linear Regression Analysis

	Time (Splines)														
	T1 (0–10 s)			T2 (10–20 s)			T3 (20–30 s)			T4 (30–40 s)			T5 (40–120 s)		
	β_1_	95% CI	*P*	β_1_	95% CI	*P*	β_1_	95% CI	*P*	β_1_	95% CI	*P*	β_1_	95% CI	*P*
sBP	−0.344	−0.707 to 0.018	0.063	0.060	−0.302 to 0.422	0.746	0.154	−0.206 to 0.516	0.400	0.034	−0.322 to 0.392	0.848	−0.037	−0.109 to 0.034	0.303
dBP	−0.302	−0.505 to −0.100	0.003^*^	0.158	−0.044 to 0.360	0.125	0.108	−0.092 to 0.309	0.291	−0.053	−0.250 to 0.142	0.591	−0.011	−0.046 to 0.022	0.502
MAP	−0.295	−0.547 to 0.043	0.022	0.102	−0.149 to 0.353	0.427	0.147	−0.102 to 0.397	0.247	−0.043	−0.289 to 0.202	0.727	−0.019	−0.065 to 0.026	0.413
HR	0.026	−0.111 to 0.165	0.706	0.262	0.124 to 0.401	<0.001^*^	−0.106	−0.244 to 0.032	0.132	0.035	−0.101 to 0.172	0.615	−0.005	−0.034 to 0.023	0.709
CO	−0.026	−0.051 to −0.001	0.040	0.026	0.001 to 0.051	0.038	−0.004	−0.029 to 0.019	0.694	0.013	−0.010 to 0.037	0.277	0.000	−0.004 to 0.004	0.941
SV	−0.364	−0.690 to −0.039	0.028	0.225	−0.098 to 0.550	0.173	0.034	−0.288 to 0.357	0.834	0.213	−0.105 to 0.533	0.190	0.004	−0.058 to 0.066	0.899
TPR	9.357	3.683 to 15.030	0.001^*^	−6.718	−12.31 to −1.118	0.019^*^	0.946	−4.627 to 6.520	0.739	−2.654	−8.160 to 2.850	0.345	−0.053	−1.137 to 1.030	0.923

Table displays beta coefficients (β_1_) for changes in hemodynamic measures associated with AMD progression (*n* = 38) compared to participants without AMD progression (*n* = 121) in the fully adjusted model (Model 3) adjusted for age, sex, BMI, smoking status (never vs. past/current), CVD burden (0 vs. 1 or 2 or ≥3), antihypertensive use, grade of AMD at baseline, family history of AMD, and carriers of the *CFH* and *ARMS2* risk variant. CI, confidence interval.

* Statistically significant.

From this fully adjusted mixed effects model (Model 3), factors that also significantly contributed to change in hemodynamic parameters during the AS are outlined in Table 3. This analysis showed significant associations between cardiovascular/neurovascular disease burden (CVD/NVD) and change in hemodynamic parameters during the AS test. In the first 10 seconds after standing, compared with having no CVD/NVD, having one or two diseases was associated with a greater drop in sBP, dBP, and MAP, as well as TPR. For TPR, this association was also significant for those with three or more diseases. During the 10- to 20-second recovery phase, having one or two CVDs/NVDs was associated with slower recovery in HR, but this effect was not observed with three or more conditions. Other associations with age, female sex, BMI, smoking, being on antihypertensives, SNPs of interest, family history of AMD, and grade of AMD at baseline are reported in Table 3.

**Table 3.** Association Between AMD-Risk Associated Variables and Hemodynamic Responses to Active Stand Test

	sBP			dBP			MAP			HR			CO			SV			TPR		
	β_1_	95% CI	*P*	β_1_	95% CI	*P*	β_1_	95% CI	*P*	β_1_	95% CI	*P*	β_1_	95% CI	*P*	β_1_	95% CI	*P*	β_1_	95% CI	*P*
0–10 s Post-Stand (Initial Drop)																					
Age (y)	−0.011	−0.031 to 0.007	0.225	0.011	0.000 to 0.021	0.043	0.001	−0.011 to 0.015	0.804	−0.017	−0.024 to −0.010	<0.001	−0.001	−0.002 to 0.000	0.071	0.021	0.003 to 0.038	0.016	−0.910	−1.212 to −0.609	<0.001
Sex (female)	−0.174	−0.475 to 0.125	0.254	0.203	0.035 to 0.371	0.017	0.178	−0.029 to 0.386	0.093	0.040	−0.074 to 0.155	0.486	−0.076	−0.096 to −0.055	<0.001	−0.829	−1.098 to −0.560	<0.001	4.483	−0.168 to 9.135	0.059
BMI	0.058	0.023 to 0.092	0.001	0.033	0.014 to 0.053	0.001	0.035	0.011 to 0.059	0.004	0.020	0.007 to 0.033	0.002	−0.000	−0.002 to 0.002	0.955	−0.026	−0.057 to 0.003	0.087	1.439	0.909 to 1.969	<0.001
Smoking (current)	0.485	−0.041 to 1.011	0.071	0.224	−0.069 to 0.518	0.134	0.304	−0.060 to 0.669	0.102	0.071	−0.129 to 0.272	0.487	0.043	0.007 to 0.079	0.018	0.405	−0.065 to 0.877	0.091	−9.758	−17.90 to −1.614	0.019
CVD/NVD (1 or 2)	−0.898	−1.352 to −0.443	<0.001	−0.601	−0.854 to −0.347	<0.001	−0.707	−1.023 to −0.392	<0.001	−0.168	−0.342 to 0.004	0.057	0.001	−0.030 to 0.032	0.944	0.284	−0.122 to 0.691	0.170	−14.113	−21.16 to −7.062	<0.001
CVD/NVD (≥3)	1.063	−0.059 to 2.185	0.063	−0.635	−1.262 to −0.008	0.047	0.069	−0.709 to 0.847	0.862	0.151	−0.276 to 0.580	0.488	0.013	−0.063 to 0.090	0.729	0.495	−0.509 to 1.500	0.334	−41.158	−58.51 to −23.79	<0.001
Antihypertensives (yes)	0.411	0.042 to 0.780	0.029	0.252	0.046 to 0.458	0.016	0.393	0.137 to 0.648	0.003	−0.213	−0.354 to −0.072	0.003	0.015	−0.009 to 0.040	0.236	0.344	0.013 to 0.674	0.041	5.789	0.038 to 11.54	0.048
AMD grade	−0.316	−0.493 to −0.138	<0.001	−0.181	−0.280 to −0.082	<0.001	−0.223	−0.347 to −0.100	<0.001	0.011	−0.056 to 0.079	0.734	0.010	−0.002 to 0.022	0.105	0.163	0.004 to 0.322	0.044	−2.258	−5.008 to 0.491	0.107
10–20 s Post-Stand (Recovery Phase)																					
Age (y)	−0.044	−0.063 to −0.025	<0.001	−0.039	−0.049 to −0.028	<0.001	−0.034	−0.047 to −0.021	<0.001	0.035	0.028 to 0.042	<0.001	0.004	0.002 to 0.005	<0.001	0.012	−0.004 to 0.029	0.151	−0.609	−0.906 to −0.311	<0.001
Sex (female)	−0. 721	−1.021 to −0.421	<0.001	−0.462	−0.630 to −0.295	<0.001	−0.501	−0.709 to −0.293	<0.001	−0.017	−0.132 to 0.097	0.770	0.053	0.032 to 0.074	<0.001	0.505	0.236 to 0.773	<0.001	−11.681	−16.31 to −7.051	<0.001
BMI	−0.003	−0.037 to 0.031	0.861	−0.016	−0.035 to 0.002	0.096	−0.011	−0.035 to 0.011	0.325	−0.003	−0.016 to 0.009	0.590	0.000	−0.001 to 0.003	0.583	0.019	−0.011 to 0.049	0.221	−0.888	−1.416 to −0.359	0.001
Smoking (current)	−0.535	−1.061 to −0.009	0.046	−0.345	−0.639 to −0.052	0.021	−0.358	−0.723 to 0.005	0.054	0.127	−0.073 to 0.328	0.213	0.008	−0.027 to 0.044	0.651	0.059	−0.411 to 0.529	0.805	−4.007	−12.12 to 4.107	0.333
CVD/NVD (1 or 2)	0.280	−0.173 to 0.734	0.226	0.185	−0.067 to 0.439	0.151	0.217	−0.097 to 0.532	0.175	0.321	0.147 to 0.494	<0.001	0.042	0.011 to 0.073	0.009	0.067	−0.339 to 0.473	0.746	−1.029	−8.035 to 5.976	0.773
CVD/NVD (≥3)	−0.640	−1.761 to 0.480	0.263	−0.451	−1.077 to 0.174	0.157	−0.470	−1.248 to 0.306	0.235	−0.092	−0.520 to 0.335	0.672	0.064	−0.012 to 0.141	0.099	0.566	−0.437 to 1.569	0.269	−1.289	−18.59 to 16.01	0.884
Antihypertensives (yes)	−0.554	−0.923 to −0.186	0.003	−0.147	−0.352 to 0.058	0.160	−0.344	−0.600 to −0.089	0.008	0.012	−0.127 to 0.153	0.857	−0.021	−0.047 to 0.003	0.094	−0.377	−0.706 to 0.047	0.025	−2.051	−7.737 to 3.633	0.479
AMD grade	0.142	−0.034 to 0.320	0.115	0.089	−0.009 to 0.188	0.077	0.093	−0.294 to 0.216	0.136	0.006	−0.061 to 0.073	0.858	−0.003	−0.015 to 0.008	0.562	−0.005	−0.164 to 0.153	0.945	0.125	−2.615 to 2.865	0.929
Main Effects of Family History of AMD: Carriers of the *CFH* rs1061170 and *ARMS2* rs10490924 Risk Variants and Hemodynamic Responses During AS Test																					
CFH (rs1061170)	2.967	−2.077 to 8.012	0.249	−0.389	−2.95 to 2.17	0.766	0.638	−2.630 to 3.907	0.702	−1.969	−3.623 to −0.316	0.020	0.051	−0.236 to 0.340	0.724	3.60	−0.892 to 8.100	0.116	−1.00	−79.83 to 77.81	0.980
*ARMS2* (rs10490924)	0.494	−3.934 to 4.924	0.827	−0.013	−2.26 to 2.23	0.990	0.219	−2.650 to 3.090	0.881	0.797	−0.654 to 2.249	0.282	0.078	−0.174 to 0.331	0.545	0.429	−3.518 to 4.377	0.831	−11.78	−80.99 to 57.43	0.739
Family history (yes)	1.463	−7.062 to 9.988	0.737	0.562	−3.76 to 4.89	0.799	0.863	−4.661 to 6.387	0.759	1.256	−1.537 to 4.051	0.378	−0.011	−0.499 to 0.475	0.963	−0.295	−7.894 to 7.303	0.939	49.56	−83.66 to 182.7	0.466

Table displays beta coefficients (β_1_) for change in hemodynamic measures associated with AMD-associated risk variables derived from the fully adjusted model (Model 3). Coefficients are associated with unit increases in the continuous variables age and BMI. For categorical variables, the beta coefficient is associated with group for sex (female vs. male), smoking (current vs. never/past), CVD (1 or 2 or ≥3 vs. 0), and antihypertensive use (yes vs. no). Main-effect coefficients and association with hemodynamic change during AS are presented for carriers of the *CFH* and *ARMS2* risk variant and family history of AMD (yes vs. no).

## Discussion

In this study, we utilized continuous hemodynamic monitoring to compare two groups of TILDA participants according to their AMD progression status over 4 years. We found significant differences in indicators of impaired hemodynamic responses, particularly in the first 20 seconds post-standing, which could be associated with the pathophysiology of AMD progression. At baseline, older age and lower dBP were significantly associated with AMD progression. Mixed effects models for the period between standing and 10 seconds post-stand revealed significant independent associations between AMD progression and a steeper drop in dBP, as well as a slower drop in TPR. A steeper drop in MAP and slower increases in SV and CO were also associated with AMD progression; however, this did not cross the threshold for significance when controlling for multiple comparisons. Between 10 and 20 seconds post-stand, AMD progression was independently associated with a less pronounced reduction in HR, and a less pronounced drop in CO and slower TPR increase were also noted. Together, these findings point toward a possible situation of lower baseline dBP and poor compensatory responses during the early phases post-orthostasis in participants with AMD progression. This could hypothetically reflect a higher risk of transient hypoxia in retinal tissues as a possible underlying pathophysiological mechanism.

The dBP represents the pressure in arteries when the heart is at rest between beats and is vital for perfusion of the heart and other end-organs, including the eye and retina.^23^^,^^24^ Participants with AMD progression had average dBP levels approximately 5 mmHg lower than participants without progression, suggesting that perfusion of sensitive end-tissues (e.g., myocardium) may be lower and contributing to a higher overall sympathetic tone observed in early hemodynamic responses in the AMD progression group, which could be interpreted as an attempt to compensate an underlying perfusion problem. Regression analysis also showed that AMD progression was associated with a greater reduction in dBP independently of risk factors associated with AMD.

When correcting for multiple comparisons, we did not observe a significant association with AMD progression and change in MAP; however, we did note a trend toward the AMD progression group having a steeper drop in MAP upon standing. It has been demonstrated that ocular perfusion pressure is dependent on MAP,^25^ and the proposed minimum required MAP to adequately perfuse organs and maintain homeostasis is around 60 mmHg.^26^ Participants with AMD progression had a drop in MAP of around 28% from baseline, falling <10 mmHg of the critical lower limit. Although this is a transient effect, older adults can stand up to 50 to 70 times a day,^27^^,^^28^ resulting in repeated low MAP exposure and inadequate perfusion. The current gold-standard treatment available to people with late-stage neovascular AMD is monoclonal antibodies targeting vascular endothelial growth factor (VEGF), a potent pro-angiogenic factor whose expression is regulated by hypoxia and found at increased levels in the eyes of AMD patients.^29^ Transient retinal hypoxia induced by low MAP exposure could be contributing to increased VEGF expression in the retina and driving AMD progression.

The use of linear splines between time points during AS allowed the rate of change in BP measures to be assessed. AMD progression was associated with a more rapid decline in sBP, dBP, and MAP compared to those with no AMD progression. This rapid drop in BP after standing has been observed in other studies, where rate of drop, more than magnitude of drop, was associated with poor physical performance, frailty, and falls in older people,^30^^,^^31^ suggesting that this presents a greater challenge to the baroreceptor reflex, impairing BP recovery.^30^

In response to a drop in BP upon standing, counteracting circulatory mechanisms are employed to maintain arterial pressure through activation of the sympathetic nervous system. These measures induce vasoconstriction aiding venous return to the heart, causing an increase in stroke volume and cardiac output.^13^^,^^32^^,^^33^ Regression analysis noted a reduced drop in TPR associated with the AMD progression group after standing, suggesting attenuated vasodilation of systemic vessels in response to activation of skeletal muscles. However, differences observed between participants with and without AMD progression were also found in measures of SV and CO. In conjunction with increasing HR, we found that CO and SV increased within the first 10 seconds of standing. For participants with no AMD progression, this effect was transient and rapidly decreased during the recovery phase. However, in those with AMD progression, we observed a slower increase in SV and CO in response to standing. This was combined with slower recovery of CO during the recovery phase. HR recovery was found to be significantly slower in the AMD progression group. This is consistent with previous observations that delayed HR recovery after standing or cessation of exercise predicts cardiovascular events and all-cause mortality.^34^^,^^35^

Several factors known to be associated with AMD were examined in this analysis, and many were found to contribute significantly to the model; however, orthostatic hemodynamic effects found to be associated with AMD progression in our study remained independent of these factors. For example, the mixed-effects analysis showed a more pronounced drop in BP and TPR associated with increasing CVD burden and grade of AMD at baseline. Increasing age is not only a substantial risk factor for AMD but also associated with CVD and autonomic impairment.^36^ In this cohort, participants with AMD progression were on average 4 years older than those with no AMD progression. Regression analyses found that attenuated HR and TPR during the initial 10 seconds post-stand were significantly associated with older age and strongly associated with almost all hemodynamic measures during the recovery phase (10–20 seconds post-stand). This indicates that age-related dysfunction of autonomic cardiovascular control may be mediated through impaired HR activation causing delayed BP recovery.

Hypertension is frequently reported as a risk factor for AMD, with sustained orthostatic hypertension previously shown to be a risk factor for AMD.^37^ In this cohort, we found that participants, on average, had high blood pressure (sBP, >130 mmHg; dBP, >80 mmHg) at baseline, with 33% taking some form of antihypertensive medication. The association between antihypertensive medication and AMD has previously been examined in several population studies with inconsistent results.^38^^–^^40^ In this study, antihypertensive use was associated with a reduced peak in HR during the initial change in hemodynamic measures at 10 seconds post-stand combined with a slower recovery in sBP and MAP at 10 to 20 seconds. Antihypertensive use was significantly higher in participants with AMD progression; therefore, the association with slower recovery in sBP and MAP during orthostasis could present an additional challenge to this group, which already displays low dBP at baseline and a significant drop in dBP during orthostasis. These features together may promote inadequate perfusion of tissues.

Although this study indicates that impaired orthostatic hemodynamic responses may be implicated in the progression of AMD, it is important to note that as an extension of the central nervous system, blood flow within the eye is autoregulated to maintained blood flow at a near constant level to meet the high metabolic demand of the eye.^41^ Autoregulation is also observed in cerebral vasculature,^42^ and studies have shown that changes in BP, particularly in orthostatic hypotension, have been associated with changes in cerebral perfusion and cerebral hypoxia, despite autoregulatory control.^43^ It remains to be determined if similar failures in autoregulation control of retinal blood flow occurs in AMD advancing its progression. However, the choroid is innervated by the sympathetic and parasympathetic nervous system and therefore susceptible to impaired autonomic control. Previous studies have found that sympathetic denervation of the choroid in a rodent model displays impaired baroregulation of choroidal blood flow in response to elevated systemic arterial BP correlating with a decline in retinal function and photoreceptor cell loss, retinal pigment epithelial atrophy, and morphological change of the choroid and Bruch's membrane,^44^^,^^45^ suggesting that impaired autonomic regulation of choroidal blood flow can have a direct impact on retinal structure and visual function.

### Limitations

This study was limited by the low number of individuals who were included relative to the main TILDA sample. This limitation arises from the expected low prevalence of AMD in a population-based study. Indeed, the TILDA cohort is a longitudinal population study on community-dwelling adults who undergo extensive questioning and physical assessment during assessment. Therefore, participants with pronounced failures in autonomic function would most likely be underrepresented in this relatively robust cohort.

## Conclusions

In conclusion, this study suggests that early orthostatic hemodynamic impairments could offer clues to the pathophysiology of AMD progression and potentially introduce new therapeutic approaches to delay the progression of AMD disease. However, further research is needed to validate this in external cohorts.

## Supplementary Material

Supplement 1

Supplement 2
