# Supplementary material for: Cardiovascular Autonomic Function and Progression of Age-Related Macular Degeneration in The Irish Longitudinal Study of Ageing (TILDA)
Source: Invest Ophthalmol Vis Sci. 2024 Jun 14;65(6):24. doi: 10.1167/iovs.65.6.24 (PMC11182369; doi:10.1167/iovs.65.6.24)
Supplement: Supplement 1 [file iovs-65-6-24_s001.pdf]

SUPPLEMENTARY TABLE 1: Change in AMD grade from wave 1 to wave 3 in TILDA participants in study cohort (n=159)

		Total		AMD Grade at W3													
				Early Mild		Early Moderate		Early Severe		Late GA		Late NV		No Disease		Late Mixed	
		**n**	**(%)**	**n**	**(%)**	**n**	**(%)**	**n**	**(%)**	**n**	**(%)**	**n**	**(%)**	**n**	**(%)**	**n**	**(%)**
**AMD GRADE AT W1**	Early Mild	68	(100)	40	(58.82)	23	(33.82)	0	0	0	0	0	0	5	(7.35)	0	0
	Early Moderate	65	(100)	7	(10.77)	45	(69.23)	10	(15.38)	1	(1.54)	0	0	2	(3.08)	0	0
	Early Severe	19	(100)	0	(0)	7	(36.84)	11	(57.89)	1	(5.26)	0	0	0	0	0	0
	Late NV	3	(100)	0	(0)	0	0	0	0	0	0	2	(66.67)	0	0	1	(33.3)
	Late GA	3	(100)	0	(0)	0	0	0	0	3	(100)	0	0	0	0	0	0
	Late Mixed	1	(100)	0	(0)	0	0	0	0	0	0	0	0	0	0	1	(100)
	**Total**	159	(100)	47	(29.56)	75	(47.17)	21	(13.21)	5	(3.14)	2	(1.26)	7	(4.40)	2	(1.26)

AMD = Age-related Macular Degeneration; AS = Active Stand test; GA = Geographic Atrophy; NV = neovascular; W1 = Wave 1; W3 = Wave 3.
