# Supplementary material for: Cardiovascular Autonomic Function and Progression of Age-Related Macular Degeneration in The Irish Longitudinal Study of Ageing (TILDA)
Source: Invest Ophthalmol Vis Sci. 2024 Jun 14;65(6):24. doi: 10.1167/iovs.65.6.24 (PMC11182369; doi:10.1167/iovs.65.6.24)
Supplement: Supplement 2 [file iovs-65-6-24_s002.pdf]

Supplementary Table 2: Wave 1 characteristics of participants who returned/did not return at wave 3.

Participants eligible for inclusion in study (n=200)		
**Retinal images taken n (%)**	**200 (100%)**	*P* Value
Male n (%)	86 (43%)	
Female n (%)	114 (57%)	
Age at W1 mean (SD)	63 (8.7)	
Seated systolic BP at W1 mean (SD)	134.9 (19.4)	
Seated diastolic BP at W1 mean (SD)	82.8 (11.2)	
Participants who are not eligible (n=100)		
**No retinal images taken n (%)**	**65 (100%)**	
Male n (%)	33 (51%)	
Female n (%)	32 (49%)	
Age at W1 mean (SD)	66 (9.7)	0.008
Seated systolic BP at W1 mean (SD)	140.6 (19.0)	0.037
Seated diastolic BP at W1 mean (SD)	82.8 (10.8)	0.962
**Passed away n (%)**	**6 (100%)**	
Male n (%)	3 (50%)	
Female n (%)	3 (50%)	
Age at W1 mean (SD)	73 (6.1)	0.006†
Seated systolic BP at W1 mean (SD)	127.6 (13.7)	0.258†
Seated diastolic BP at W1 mean (SD)	74.2 (7.0)	0.028†
**Did not take part in wave n (%)**	**29 (100%)**	
Male n (%)	14 (48%)	
Female n (%)	15 (52%)	
Age at W1 mean (SD)	66 (9.8)	0.115
Seated systolic BP at W1 mean (SD)	140.8 (23.7)	0.214†
Seated diastolic BP at W1 mean (SD)	85.4 (16.0)	0.392†

W1= wave 1; W3= wave 3; CAPI = computer aided personal interview; BP= Blood pressure

† P value calculated from comparison with participants who had retinal images taken (n=200).
